# Syk inhibitors in clinical development for hematological malignancies

**DOI:** 10.1186/s13045-017-0512-1

**Published:** 2017-07-28

**Authors:** Delong Liu, Aleksandra Mamorska-Dyga

**Affiliations:** 1 Department of Oncology, The first Affiliated Hospital of Zhengzhou University, Zhengzhou, 450052 China; 2 Department of Medicine, New York Medical College and Westchester Medical Center, Valhalla, NY 10595 USA

## Abstract

Spleen tyrosine kinase (Syk) is a cytosolic non-receptor protein tyrosine kinase (PTK) and is mainly expressed in hematopoietic cells. Syk was recognized as a critical element in the B-cell receptor signaling pathway. Syk is also a key component in signal transduction from other immune receptors like Fc receptors and adhesion receptors. Several oral Syk inhibitors including fostamatinib (R788), entospletinib (GS-9973), cerdulatinib (PRT062070), and TAK-659 are being assessed in clinical trials. The second generation compound, entospletinib, showed promising results in clinical trials against B-cell malignancies, mainly chronic lymphoid leukemia. Syk inhibitors are being evaluated in combination regimens in multiple malignancies.

## Background

Spleen tyrosine kinase (Syk) is a cytosolic non-receptor protein tyrosine kinase (PTK) which was discovered in 1990 [1]. The full-length *syk* cDNA was first cloned from porcine spleen cells in 1991 [2]. The human *syk* cDNA contains an open reading frame of 1908 base pairs. The human *syk* gene encodes a 635-aa polypeptide with an estimated molecular weight of 72 kDa [3, 4]. The syk gene was found to be localized on chromosome 9q22. Syk is mainly expressed in hematopoietic cells. Syk belongs to the Src family of non-receptor type PTKs and is highly homologous to ZAP-70, which is thought to be the Syk counterpart in T cells [4, 5].

Syk contains two N-terminal SH2 domains and one C-terminal tyrosine kinase domain. The SH2 domains of Syk bind to immunoreceptor tyrosine-based activation motifs (ITAMs), leading to Syk activation. Syk protein lacks myristoylation site, therefore does not attach directly to the cell membrane [2, 4, 5].

Syk has an autophosphorylation site at Tyr-518. Following receptor engagement such as antigen binding or sIgM ligation in B cells, tyrosine residues are phosphorylated by Lyn, another Src-family non-receptor PTK. The phosphorylation on the tyrosine residues in Syk creates binding sites for CBL, VAV1, and phospholipase C-gamma, the regulators of B-cell receptor (BCR) signaling pathways. These lead to an increase in second messenger IP3 which stimulates calcium ion mobilization.

## Syk functions

Syk was recognized as a critical element in the BCR signaling pathway [6, 7]. Syk is also a key component in signal transduction from other immune receptors like Fc receptors and adhesion receptors. Syk along with other BCR signaling molecules, Bruton tyrosine kinase (BTK), PI3K delta (PI3Kδ), and tumor necrosis factor (TNF) superfamily receptors was also found to be involved in signal transduction independent from the BCR [8–10]. Syk is expressed primarily in hematopoietic cells like B-cells, monocytes, macrophages, mast cells, and neutrophils. Syk was recognized to be a potential target for the treatment of various hematologic cancers, autoimmune disorders, and other inflammatory states [11–16]. Under resting conditions, Syk remains in unphosphorylated state. Activation of the BCR leads to oligomerization and phosphorylation of the Igα and ß (immunoreceptor tyrosine-based activation motifs, ITAMs), the transmembrane signaling proteins CD79a and CD79b. The latter results in activation of the Syk tyrosine kinase, which in turn initiates downstream signaling through PI3K and BTK leading to amplification of the original BCR signal. In animal models Syk was found to be a critical point in B-cell antibody responses, differentiation into germinal center or plasma cells and memory B-cells [17–19].

## Syk inhibitors

Several oral Syk inhibitors including fostamatinib (R788), entospletinib (GS-9973), cerdulatinib (PRT062070), and TAK-659 are being assessed in clinical trials [9]. Preclinical studies of a few Syk inhibitors have been reported [20–23].

### Fostamatinib

Fostamatinib is the first oral Syk inhibitor (previously known as R788). It is rapidly metabolized in vivo to R406 [24, 25]. Fostamatinib can selectively abrogate the BCR signaling pathway. Fostamatinib has potent anti-inflammatory effects [18, 26]. It was first demonstrated to have activities in rheumatoid arthritis and immune thrombocytopenia [11, 27–31]. In a murine model of chronic lymphoid leukemia (CLL) fostamatinib was found to induce an early and transient mobilization of both normal and malignant B cells, but selectively inhibited the growth of the malignant B-cell population [32]. This effect on mobilization of B cells is similar to that of BTK inhibitors [10, 33–39].

Fostamatinib was the first clinically available Syk inhibitor which was tested in a phase I/II study in patients with refractory B-cell lymphomas [40]. The dose-limiting toxicity (DLT) was diarrhea, neutropenia, and thrombocytopenia. The highest response (*n* = 6, 55%) was seen in patients with CLL/SLL (*n* = 11). The overall response rate (ORR) among the 23 patients with diffuse large B-cell lymphoma (DLBCL) in the phase II part was 22%. Among the five responders, there were one complete response (CR) and four partial responses (PR). Median progression-free survival (PFS) for the patients in the phase II group was 4.2 months, and for DLBCL patients, the median PFS was 2.7 months. This trial established the phase II dose at 200 mg BID.

Fostamatinib was studied in patients with relapsed or refractory DLBCL in a phase II, two-arm, randomized, double-blinded trial at either 100 mg twice a day (BID) or 200 mg BID. The ORR was the primary endpoint. Due to limited efficacy at the lower dosage, all subsequent patients were treated at 200 mg BID. At the time of the report [41], 68 patients were treated (47 at 200 mg BID, 21 at 100 mg BID). Of these patients, 58% were germinal B-cell (GCB) origin, 30% activated B-cell (ABC) origin. Diarrhea, nausea, and fatigue were the most common treatment-related adverse events (TEAE). The ORR was 3%. Patients with the ABC genotype failed to respond. Off-target effects on VEGFR and other tyrosine kinases were thought to be responsible for the most common TEAEs [24, 42].

### Entospletinib (GS-9973)

The search for more kinase-selective oral Syk inhibitor resulted in the discovery of GS-9973, entospletinib [43]. This second generation compound demonstrated favorable in vitro and in vivo selectivity profile with fewer dose-limiting adverse effects. Entospletinib in comparison to fostamatinib revealed increased selectivity for Janus kinase 2 (JAK-2), c-KIT, FMS-like tyrosine kinase 3 (FLT 3), RET, and VEGFR2 [43].

Preclinical studies in CLL cell lines demonstrated that inhibition of Syk activity leads to decreased secretion of CCL3 and CCL4 resulting in cell redistribution. The disruption of the microenvironment of B-cells and surrounding stroma by Syk inhibitors mitigates the chemokine- and integrin-mediated protective effects of CLL cells [44–46]. Clinically this effect manifests as lymph node reduction with associated transient marked lymphocytosis. These effects make entospletinib an ideal agent for refractory CLL and other B cell non-Hodgkin lymphomas (NHL) [47, 48].

Entospletinib (GS-9973) was evaluated in a multicenter, phase 2 study on subjects with relapsed or refractory CLL (*n* = 41) and non-Hodgkin lymphoma (*n* = 145) [47]. Patients received 800 mg entospletinib twice daily. This report provided results of efficacy in CLL and safety in all patients (*n* = 186). The prior treatment for CLL must have included at least 2 cycles of therapeutic antibody or cytotoxic therapy. The median progression-free survival (PFS) for CLL was 13.8 months (95% CI, 7.7 months to not reached). The ORR was 61.0% (95% CI, 44.5–75.8%), with 25 partial responses (PR), 13 patients with stable disease, and no complete responses (CR). 94.9% of the patients in the CLL cohort achieved reduction in adenopathy. There were three patients who had nodal reduction with persistent lymphocytosis. The rate of serious adverse events (SAEs) was 29%. The most common TEAEs included dyspnea, pneumonia, febrile neutropenia, and pyrexia. The study showed that entospletinib had clinical activity in subjects with relapsed or refractory CLL [49]. The toxicity was acceptable in the whole patient population with CLL and NHL.

In updated report of 69 patients with indolent NHL (41 follicular lymphoma (FL), 11 lymphoplasmacytoid lymphoma (LPL), 17 marginal zone lymphoma (MZL)), entospletinib 800 mg BID was evaluated in a multicenter phase II study as mentioned above [48]. Median age of the cohorts was 66 years (range 41–89). The subjects received a median number of 3 prior treatment regimens (range 1–14). Entospletinib was generally well tolerated. The most common TEAEs were fatigue and GI toxicities. Common laboratory abnormalities were elevation of ALT /AST and bilirubin as well as anemia and neutropenia. The ORR was 13.0% (95% CI: 6.1% to 23.3%), with 7 PR, and one CR. The median PFS was 5.5 months (95% CI: 4.4 to 8.2 months). Therefore, entospletinib monotherapy 800 mg BID was well tolerated and showed clinical activity in patients with advanced indolent NHL. Entospletinib was shown recently in a phase II study to have modest activity in 39 patients with relapsed or refractory mantle cell lymphoma (MCL) [50]. Further development of entospletinib in NHL will likely focus on the combination regimens [50].

Recently, the interim results of the entospletinib monotherapy and in combination with chemotherapy in patients with AML were presented. In this ongoing phase 1b/2 study (NCT02343939), entospletinib was well tolerated [51]. At the time of this report, the study enrolled 12 patients with a median age of 54 (range, 18–69) years. Three patients were treated at 200 mg BID, and six were dosed at 400 mg BID. The most common non-hematologic AEs included febrile neutropenia, nausea, and diarrhea. Taken all the data together, there was lack of benefit to escalate dose pass 400 mg BID. Therefore, 400 mg BID was selected as the recommended phase 2 dose. Surprisingly, 9/9 patients (100%) treated at both levels achieved CR. One patient with 11q23-rearranged AML achieved morphologic and cytogenetic CR after 14 days on entospletinib monotherapy, indicating unique sensitivity to the drug in this patient with poor prognosis [51].

### Entospletinib in combination regimens

PI3Kδ inhibitors and BTK inhibitors as well as immune check point inhibitors are novel agents that have changed the clinical management of CLL and NHLs [8, 10, 35, 52–56]. Prolonged administration of these agents is, however, needed, and resistance is already emerging in a proportion of patients [57, 58]. Therefore, combination of entospletinib and idelalisib was evaluated in an attempt to enhance the efficacy and reduce resistance [59, 60].

An open-label phase II study evaluated the safety and efficacy of the combination of entospletinib and idelalisib [60]. The study included 66 patients in five cohorts of indolent NHL, CLL, MCL, and DLBCL. The primary end point was ORR. The patients underwent intra-patient dose escalation of idelalisib and entospletinib (from 100 to 150 mg and 200 to 800 mg twice daily, respectively) every 2 or 4 weeks. After treatment for a median of 10 weeks, the ORR reached 60% in the CLL cohort, 36% in follicular lymphoma, and 17% in DLBCL. The most common AEs were diarrhea, rash, and transaminitis, which improved after treatment discontinuation. However, there were 12 patients who developed pneumonitis, 11 of grade 3 or more, resulting in two fatalities. The study was therefore terminated. Such a severe pro-inflammatory response was attributed to the unexpected potent inhibition of the mammalian target of rapamycin (mTOR) observed with the combination of the two study drugs. Increased T helper 1 (Th-1) response mediated by interferon-γ, IL- 6, 7, 8 was reported in patients with pneumonitis [60].

Events from the idelalisib and entospletinib phase II study prompt researchers to exert caution in planning and conducting further trials with Syk inhibitors in conjunction with other BCR inhibitors or in combination with chemotherapy (Table 1).

**Table 1:** Clinical trials of entospletinib

Phase	Diseases	Interventions	NCT ID
2	CLL, MCL, DLBCL, non-FL iNHL, FL	Entospletinib monotherapy	NCT01799889
1/2	AML	Entospletinib monotherapy	NCT02343939
2	CLL, MCL, DLBCL, iNHL	Entospletinib with idelalisib	NCT01796470
1	ALL	Entospletinib, vincristine, dexamethasone	NCT02404220
2	GVHD	Entospletinib, placebo, corticosteroids	NCT02701634
1		Entospletinib	NCT02521376
1	B-cell malignancies	Entospletinib, idelalisib, ONO/GS-4059	NCT02457598
1, 2	NHL	Entospletinib, vincristine	NCT02568683

*AML* acute myelogenous leukemia, *ALL* acute lymphocytic leukemia, *CLL* chronic lymphocytic leukemia, *DLBCL* diffuse large B-cell lymphoma, *FL* follicular lymphoma, *GVHD* graft versus host disease, *MCL* mantle cell lymphoma, *NHL* non-Hodgkin lymphoma, *iNHL* indolent NHL

Despite recent advances in molecular diagnosis and clinical therapy, relapsed and refractory acute lymphoid leukemia (R/R ALL) remains a significant challenge [61–68]. Novel agents are being developed to improve the outcome of R/R ALL [64, 65, 69–74]. Entospletinib was studied in combination with vincristine and shown to have synergistic activity in vitro in 19 hematological cancer cell lines including lymphoma, multiple myeloma, ALL, and AML [75]. The in vivo efficacy of entospletinib and vincristine as singe agents and in combination was further evaluated in a lymphoma xenograft model using the SU-DHL-10 cell line. The combination of vincristine with entospletinib showed synergistic activity in ALL in the mouse model and thus supported the further clinical development of entospletinib in combination with vincristine [76]. A phase 1b trial (NCT02404220) is evaluating entospletinib in combination with chemotherapy (vincristine and dexamethasone) for the treatment of R/R Pre B-ALL. In the last update, three patients have been enrolled [77].

### Cerdulatinib (PRT062070)

Cerdulatinib is a dual inhibitor of Syk and JAK 1/3 and has been shown to have activity against DLBCL in in vitro studies [78]. Cerdulatinib is an oral kinase inhibitor against Syk and JAK. It has been shown in vitro to have specific inhibitory activity against Syk and JAK1/3 in a subset of B-cell lymphoma cell lines. Cerdulatinib suppressed inflammation and autoantibody generation and blocked B-cell activation in a rat model and splenomegaly in a mouse model of chronic B-cell antigen receptor stimulation. In both ABC and GCB lymphoma cell lines, cerdulatinib induced apoptosis and cell-cycle arrest. Cerdulatinib blocked JAK/STAT and BCR signaling in primary GCB and non-GCB DLBCL tumor cells [79]. Primary CLL cells from patients were treated in vitro with cerdulatinib alone or in combination with venetoclax. Cerdulatinib inhibited BCR- and IL4-induced downstream signaling in CLL cells and reduced CCL3/CCL4 production. Cerdulatinib and venetoclax have synergistic activity in primary CLL cells [80]. Interestingly, cerdulatinib was shown to be better than ibrutinib in overcoming the stromal support and blocking proliferation of ibrutinib-resistant primary CLL cells and of BTKC481S-transfected/ibrutinib-resistant lymphoma cells [81]. Phase I/II dose escalation study in CLL/SLL/NHL is underway (NCT01994382) [78, 82].

### TAK-659

Syk directly binds to and activates FMS-like tyrosine kinase 3 (FLT-3) [17]. Through structure-based drug design, a novel series of heteroaromatic Syk inhibitors was screened and optimized, resulting in the discovery of TAK-659, a dual Syk and FLT3 inhibitor [83]. TAK-659 inhibited the pro-survival, proliferative, chemoresistant, and activation effects promoted by the microenvironment. Combination of TAK-659 with other BCR inhibitors showed synergistic effect in inducing apoptosis. Combination of TAK-659 and ibrutinib induced significantly higher cytotoxicity toward CLL cells [84].

TAK-659 suppressed splenomegaly and tumor development in a LMP2A/Myc mouse model in nanomolar concentrations. In addition, TAK-659 also blocked metastasis of tumor cells into bone marrow. Interestingly, TAK-659 killed tumor cells without damaging host cells in spleen and tumors [85].

In a phase 1 first-in-human study of TAK-659 (NCT02000934), patients (pts) aged ≥18 years with advanced solid tumors or lymphoma received oral TAK-659 daily (QD, 60–120 mg) [86]. At the data cutoff on June 15, 2015, 24 pts had been enrolled (14 solid tumor pts; 10 lymphoma pts) at four dose levels of TAK-659 (60, 80, 100, 120 mg). Among the 10 lymphoma pts, 7 had DLBCL (5 GCB and 2 ABC subtypes) and 3 had follicular lymphoma (FL). Among all the pts, the most common AEs were fatigue, elevated AST, anemia, and diarrhea. Eight patients (33%) had grade ≥3 drug-related AEs. Of 7 response-evaluable DLBCL pts, 3 achieved PR. Therefore, TAK-659 at dose 60–100 mg QD appears to be acceptable for further studies.

In the updated report of this phase I first-in-human study, 54 adult patients with advanced solid tumors/lymphoma received oral TAK-659 QD at doses of 60, 80, 100, and 120 mg during dose escalation (*n* = 35) or 100 mg during dose expansion (*n* = 19) [87]. There were five dose-limiting toxicities (DLTs): increased lipase levels, generalized edema, mucositis, and increased AST. MTD (maximal tolerated dose) was 100 mg daily. In this updated report, 24 DLBCL pts were evaluable for responses. Among these 24 pts, 11 (46%) achieved an objective response; (7 (29%) CR, 4 PR); all 3 response-evaluable FL pts achieved PR. No solid tumor patients achieved an objective response.

A phase Ib/II study of TAK-659 is underway in pts with relapsed or refractory acute myelogenous leukemia (R/R AML) [88]. During the phase Ib dose escalation study, adult pts with R/R AML received oral TAK-659 daily at doses of 60, 100, 120, and 160 mg. The pharmacodynamic effect of TAK-659 was assessed using flow cytometry by measuring the phosphorylation of ribosomal protein S6 (pS6) in peripheral AML blasts. DLT has not been observed in the latest update. The median age of the patients was 67 years (range 25–86), and 38% had received ≥4 prior lines of therapy. TEAEs were seen in 12 (92%) pts overall; the most common AEs were elevated AST (31%), ALT (23%), and amylase levels (23%). For PD studies, pS6 was detected at baseline and reduced after dosing in four patients (2 FLT-3-ITD; 2 FLT-3-WT). Inhibition of FLT-3-ITD phosphorylation and early activity with decreases in peripheral myeloblasts was observed. The unique mechanism of action with dual inhibition of Syk and FLT-3 appears to warrant further studies in R/R AML patients with FLT-3 mutations.

## Conclusion

Syk inhibitors are a new group of small molecule inhibitors targeting BCR-mediated signaling pathways. The second generation compound, entospletinib, showed promising results in clinical trials against B-cell malignancies, mainly CLL. There is an unmet need in hematological malignancies for the new non-chemotherapy drugs. Multidrug regimens in combination with other small molecule inhibitors and new antibodies may have the potential of overcoming or delaying the development of drug resistance [89–95]. The off-target effects of the small molecule inhibitors are a limiting factor. Caution should be exerted especially in testing drug combinations [60].

## Abbreviations

ALK: Anaplastic lymphoma kinase
BTK: Bruton tyrosine kinase
cfDNA: Cell free DNA
EGFR: Epidermal growth factor receptor
